# Arginine methylation of the p30 C/EBPα oncoprotein regulates progenitor proliferation and myeloid differentiation

**DOI:** 10.1016/j.isci.2024.111199

**Published:** 2024-10-18

**Authors:** Linh T. Nguyen, Karin Zimmermann, Elisabeth Kowenz-Leutz, Dorothea Dörr, Anja Schütz, Jörg Schönheit, Alexander Mildner, Achim Leutz

**Affiliations:** 1 Max-Delbrück Center for Molecular Medicine in the Helmholtz Association (MDC), Robert-Rössle-Street 10, 13125 Berlin, Germany; 2 BSIO Berlin School of Integrative Oncology, Charité – Universitätsmedizin Berlin, Corporate Member of Freie Universität Berlin and Humboldt-Universität zu Berlin, Berlin, Germany; 3 Institute of Biomedicine at University of Turku, Turku, Finland; 4 InFLAMES Research Flagship, University of Turku, 20014 Turku, Finland

**Keywords:** biochemistry, cell biology, transcriptomics

## Abstract

The transcription factor CCAAT enhancer binding protein alpha (C/EBPα) is a master regulator of myelopoiesis. *CEBPA* encodes a long (p42) and a truncated (p30) protein isoform from a single mRNA. Mutations that abnormally enhance expression of p30 are associated with acute myelogenous leukemia (AML). We show by mutational analysis that three highly conserved arginine residues in the p30 C/EBPα N-terminus, previously found to be methylated, are involved in myeloid lineage commitment, progenitor proliferation, and differentiation. The conservative amino acid substitution with lysine that retains the amino acid side chain charge enhanced progenitor proliferation, while a non-conservative substitution with uncharged side chains (alanine, leucine) impaired proliferation and enhanced granulopoiesis. Analysis of protein interactions suggested that arginine methylation of p30 C/EBPα differentially determines interactions with SWI/SNF and MLL complexes. Pharmacological targeting of p30 C/EBPα arginine methylation may have clinical relevance in myeloproliferative and inflammatory diseases, in neutropenia, and in leukemic stem cells.

## Highlights

• C/EBPα p30 dependent myelopoiesis is regulated by arginine methylation (Rme)
• p30 mutations mimicking Rme promote myeloid differentiation
• p30 mutation mimicking un-methylated R support proliferation
• p30 Rme affects interactions with MLL and SWI/SNF complexes

## Graphical abstract

**Figure:**
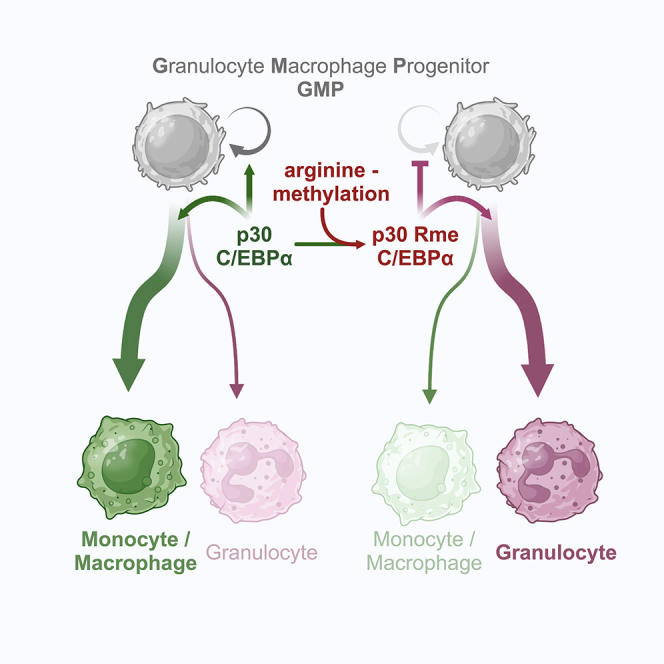

## Introduction

The C/EBPα transcription factor (TF) is an essential regulator of hematopoietic stem cell and progenitor biology. C/EBPα regulates myeloid lineage commitment, establishes the innate immune system from hematopoietic stem cells (HSC) and multipotential progenitors (MPP) and is a crucial factor in myeloid leukemogenesis. Dysregulated expression and mutations of the *CEBPA* gene are associated with acute myelogenous leukemia (AML) and neutropenia.^1^^,^^2^^,^^3^^,^^4^

*CEBPA* is a single-exon gene and produces a single mRNA, which encodes two divergent protein isoforms, the full length p42 and the truncated p30 C/EBPα. Translation of these variants can be initiated from alternative start sites in the same mRNA reading frame through the regulatory function of a small upstream open reading frame (uORF), located in the 5′ region of the mRNA.^5^ Both isoforms induce myeloid lineage commitment of MPPs, yet p42 C/EBPα causes terminal cell differentiation and cell-cycle arrest while p30 C/EBPα sustains progenitor proliferation.^6^

Approximately 7–15% of AML patients carry mutations in the *CEBPA* gene that enable expression of p30 C/EBPα but abrogate expression or function of wild type (WT) full length p42 C/EBPα. The function of dysregulated p30 C/EBPα as a driver oncoprotein was experimentally confirmed by targeted mouse genetics.^7^ Mice and early progenitor cells that is deficient for both, p42 and p30 C/EBPα, lack granulocyte macrophage progenitors (GMP) and neutrophils, and are resistant to experimentally induced AML.^8^^,^^9^ Expression of only the truncated p30 C/EBPα isoform from the murine *Cebpa* gene locus rescues GMP commitment and causes expansion of a myeloid progenitor population with complete leukemogenic penetrance.^10^ Thus, the p42 and p30 C/EBPα isoforms display overlapping and antagonistic functions balancing myelopoiesis but the precise mechanism by which p30 encompasses both, lineage commitment and leukemogenic conversion remains elusive.

Both p42 and p30 C/EBPα isoforms contain identical C-terminal basic DNA binding and leucine-zipper dimerization domains (bZip) and bind to the same regions in genomic DNA.^11^ What distinguishes the two C/EBPα isoforms is that p30 lacks parts of the p42 N-terminal tripartite transactivation domain (TAD, consisting of transregulatory elements TE1-TE3) and retains only TE3. Both C/EBPα isoforms interact with a plethora of proteins and protein complexes, but surprisingly, their interactomes are not only quantitatively but also qualitatively distinguished, suggesting discrete regulatory capacity.^12^^,^^13^

The N-terminal regions of all CEBP-family members feature intrinsically disordered regions (IDR), comprising assemblies of conserved short linear motifs (SLiM) and molecular recognition elements (MoREs) that serve as multivalent combinatorial protein-protein interaction (PPI) modules to define their interactomes. The PPI modules and the flexible linker regions contain a plethora of post-translational modifications (PTM) that kaleidoscopically fine-tune the interactomes and determine the resulting C/EBP biology.^12^^,^^14^^,^^15^ In C/EBPα, acetylation of specific lysine residues, for example K298 and K302, affects granulopoiesis while methylation of R35 directs the velocity of chromatin re-modeling and the acquisition of alternative myeloid cell fates.^16^^,^^17^

Previously, we showed that the side chains of three highly conserved arginine residues in the N-terminus of p30 C/EBPα (human R142, 149, 156; here, rat R140, 147, 154) are targets of methylation and affect the protein interactome.^12^ Here, we used mutational analysis to examine whether an amino acid side chain exchange affects p30 C/EBPα biology. Our data suggest that PTMs of arginine side chains in p30 C/EBPα critically regulate the isoform’s dichotomous activity in lineage commitment and progenitor expansion and may thus suite as future therapeutic targets.

## Results

### Transdifferentiation capacity of p30 C/EBPα

**Figure 1:**
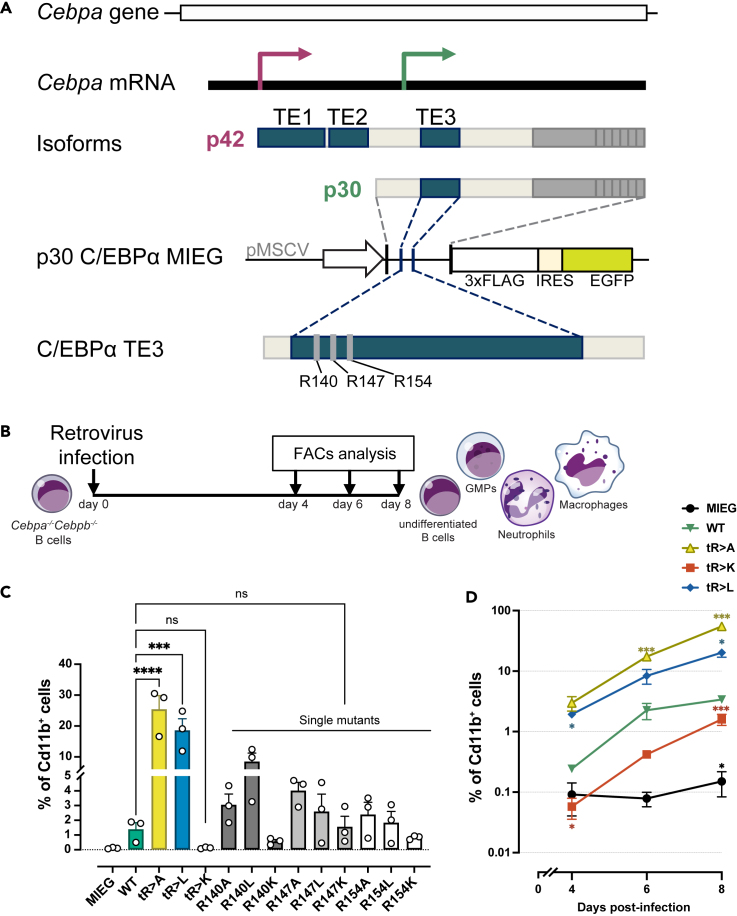
Lymphoid-myeloid transdifferentiation by WT and mutant p30 C/EBPα (A) Overview of the C/EBPα gene, mRNA, protein isoforms, and p30 C/EBPα expression constructs used in this study. The *trans*-regulatory elements (TEs) and the critical arginine residues R140, 147, 154 are depicted. The empty vector pMSCV-IRES-EGFP (MIEG) was used as control. Three arginine residues (R) of interest are shown. (B) Schematic representation of the LMT assay. *Cebpa*^*−/−*^*Cebpb*^*−/−*^ v-Abl transformed B cells (dKO-B cells) were retrovirally infected with constructs of interest. Aliquots of cell pools were harvested at indicated time points and analyzed by flow cytometry. (C). Percentage of transdifferentiated myeloid CD11b^+^ cells induced by single and triple mutants after 6 days post-infection (pi). Data are shown as mean ± SEM, significance was determined by one-way ANOVA analysis followed by Dunnett’s multiple comparisons test, *n* = 3, significance between WT p30 C/EBPα and mutants are shown. ∗*p* ≤ 0.05, ∗∗*p* ≤ 0.01, ∗∗∗*p* ≤ 0.005, ∗∗∗∗*p* ≤ 0.001. (D) Percentage of CD11b^+^ cells at various time points. Data are shown as mean ± SEM, significance was determined by two-way ANOVA analysis followed by Dunnett’s multiple comparisons test, *n* = 4. Significance of comparisons to WT p30 C/EBPα and mutants are shown by asterisks in matching color, ∗*p* ≤ 0.05, ∗∗*p* ≤ 0.01, ∗∗∗*p* ≤ 0.005, ∗∗∗∗*p* ≤ 0.001.

The truncated isoform p30 C/EBPα is implicated in myeloid cell lineage commitment, early GMP expansion, and leukemogenicity.^6^^,^^7^^,^^13^ Figure 1A depicts an overview of the C/EBPα isoforms, the critical arginine residues R140, 147, 154 reside within *trans*-regulatory element III (TEIII), and the retroviral gene transfer constructs used in this study. A lymphoid-myeloid transdifferentiation (LMT) system was employed as a versatile tool to examine the relationship of C/EBP structure and function. The LMT system is based on v-Abl transformed mouse pre-B cells that are deficient for *Cebpa* and *Cebpb* genes (dKO-B, also termed B cells in the text), which was previously shown to prevent experimental complications due to cross regulation with endogenous *Cebp* genes.^18^^,^^19^ We infected dKO-B cells with retroviral pMSCV-based EGFP-tagged p42 or p30 C/EBPα constructs. Cells expressing EGFP (GFP^+^) were then examined by cytofluorometric analysis of CD11b surface antigen expression as an indicator of myeloid transdifferentiation (see further) and loss of the B cell marker CD19 (data not shown) to monitor conversion into myeloid cells, as schematically depicted in Figures 1B and S1A.

As shown in Figures 1C and S1B, LMT conversion occurred quickly and profoundly after expression of p42 C/EBPα and slowly and sparsely with p30 C/EBPα, but not in controls. Cells that were transdifferentiated by p42 C/EBPα disappeared after 4–8 days due to arrest of proliferation, whereas p30 C/EBPα transdifferentiated myeloid cells persisted and accumulated over time (Figures S1B and S1C). These data confirm the capacity of p30 C/EBPα to induce myeloid lineage commitment without showing the growth inhibitory effect of p42 C/EBPα.

Next, we explored whether the three consecutive N-terminal arginine residues in p30 C/EBPα, previously found to be methylated, were involved in transdifferentiation and proliferation control. The arginine residues R140, R147, R154 of p30 C/EBPα were replaced individually or in combination by alanine (A), leucine (L), or lysine (K). The effect on transdifferentiation of WT and mutant p30 C/EBPα was then compared, as shown in Figure 1C. In comparison to WT p30 C/EBPα, the triple mutants, tR > A and tR > L p30 C/EBPα, strongly enhanced the conversion into myeloid CD11b^+^ cells, while transdifferentiation by the triple tR > K mutant was impaired. The single site p30 C/EBPα mutants revealed that individual arginine side chain exchanges already, yet incrementally, altered transdifferentiation, in line with the stronger effects exerted by the triple mutants. Although transdifferentiation by tR > K p30 was impaired, prolonged cultivation of tR > K p30 C/EBPα infected cells still stably maintained a small population of CD11b^+^ myeloid cells, as shown in Figure 1D.

The data suggest that the lineage commitment and differentiation capacity of p30 C/EBPα is gradually regulated by modifications of three N-terminal arginine residues. Overall, the triple mutants strongly augmented the phenotypes, as seen by the individual amino acid replacements, suggesting that the three respective side chains function in co-operation.

### Transcriptomic profiling of p30 C/EBPα transdifferentiated cells

**Figure 2:**
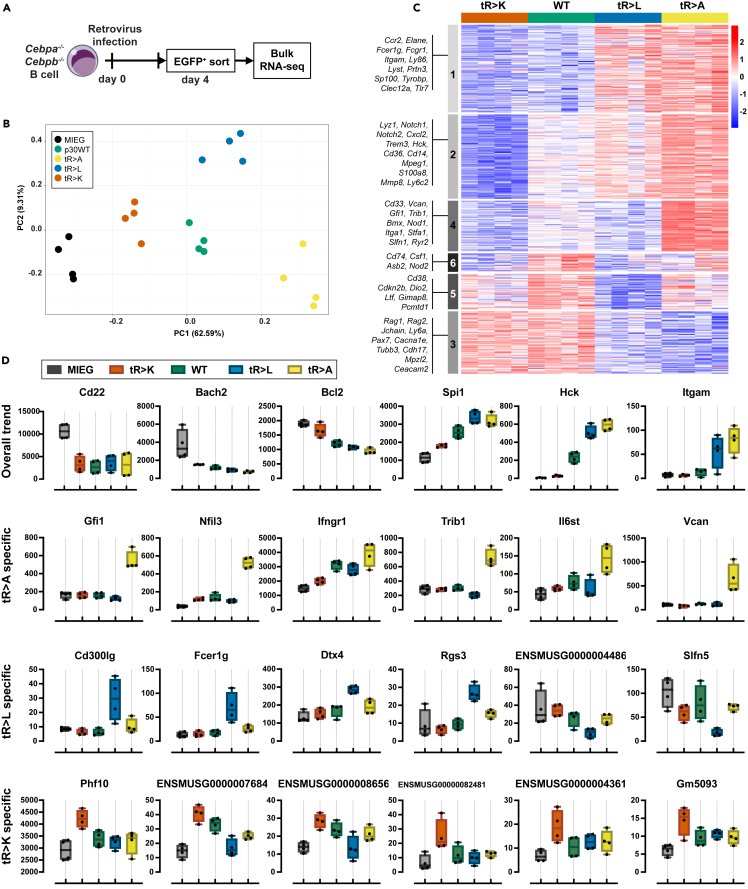
Transcriptomic profiling of WT and mutant p30 C/EBPα cells (A) Schematic representation of sample preparation for bulk RNA-seq. (B) Principal component analysis of the top 500 differentially expressed genes. Constructs are color coded and shown in inset on the left. (C) Heatmap presenting differential gene expression by constructs as indicated on the top. Color code as in B. Genes with adjusted *p* value ≤ 0.05, |FC|>2 in at least one comparison are shown. Representative gene names of each cluster are listed on the left. (D) Expression of representative genes with adjusted *p* value ≤ 0.05, |FC|>2, manually selected based on read counts. Top row: genes expressed in overall trends, last 3 rows: genes expressed in mutant-specific patterns. Data are shown as mean ± SEM

To further delineate the cellular characteristics of transdifferentiated cells generated by WT and mutant variants of p30 C/EBPα, we isolated GFP^+^ cells at day 4 post-infection (p.i.) and subjected them to bulk RNA sequencing analysis (Figure 2A). Transcriptomic expression of the C/EBPα constructs was in a same range with negligible difference (Figure S2A). Principal-component analysis (PCA) demonstrated distinct differences between WT, the p30 C/EBPα triple mutant variants, and the control group (Figure 2B), indicating that all p30 C/EBPα variants entail the capability to induce distinct cellular identities. In comparison to B cell controls, the cells expressing tR > A and tR > L p30 C/EBPα exhibited the most pronounced transcriptomic alterations, while the WT p30 C/EBPα occupied an intermediate state, between tR > A, tR > L on the one hand and tR > K p30 C/EBPα expressing cells on the other (Figure 2B).

Differential myeloid transdifferentiation processes became evident by the enrichment of gene ontology (GO) terms associated with myelomonocytic features, such as chemotaxis and the production of interleukin (IL)-1 or IL-6. Notably, residual transdifferentiation capacity was observed in the tR > K p30 C/EBPα variant, as evidenced by the GO term “monocyte chemotaxis” (Figure S2B).

Pairwise examination of differential gene expression identified 1273 genes that exhibited significant alterations (adjusted *p* value 0.05, |FC|>2) in at least one of the comparisons (Figure S2C). The global gene expression profile was consistent with the findings obtained by flow cytometric analysis of the myeloid marker CD11b (Figure 1C). Specifically, B cell-associated genes (*Cd22*, *Bach2*, and *Bcl2*) exhibited high expression levels in vector control cells, slightly reduced expression trend in p30 tR > K-expressing cells, followed by p30 WT-, tR > L-, and strong reduction in tR > A-expressing cells (Figures S2C and 2C, cluster 3). Conversely, the expression of characteristic myeloid genes, encompassing direct C/EBPα target genes, transcription factors, surface markers, and cytokines, exhibited an inverse correlation. Myeloid gene expression was highest in cells expressing the tR > A and tR > L p30 C/EBPα mutants, less pronounced in p30 WT-expressing cells, and lowest in tR > K p30 C/EBPα expressing cells (Figures S2C and 2C, cluster 1, 2).

Comparisons between WT p30 C/EBPα and each of the triple mutant variants revealed noteworthy distinctions, as shown in the cluster analysis in Figure 2C. While all three p30 C/EBPα variants WT, tR > A, and tR > L demonstrated the capacity to induce myeloid lineage switching, the tR > A and tR > L expressing cells exhibited a higher abundance of genes associated with granulocytes/neutrophils (*Elane*, *Prtn3*, *S100a8*, *Mmp8*, *Gfi1*) compared to WT p30 C/EBPα (Figure 2C, particularly clusters 1, 2, 4). On the other hand, clusters 5 and 6 represent sets of genes that were either marginally or not induced by the tR > L p30 C/EBPα mutant (Figure 2C, cluster 5, 6). These included genes that are specific to monocytes (cluster 6) and a collection of mitochondrial pseudogenes of unknown function (cluster 5). In contrast, both the tR > K and WT p30 C/EBPα variants exhibited a higher expression of genes specific for B cells (*Rag1/2*, *Jchain*, *Ly6a*), depicting the attenuated capacity of tR > K p30 C/EBPα to induce myeloid lineage switching.

Next, we searched for mutant-specific differentially expressed genes play a role in myelopoiesis but deviated from the overall trends. The tR > A and tR > L p30 C/EBPα mutants exhibited upregulation of the known C/EBPα target genes, *Trib1* and *Dtx4*, respectively. Furthermore, they augmented expression of myeloid transcripts, including *Gfi1*, *Flt3*, and *Vcam* in the case of tR > A, and *CD300lg*, *Fcer1g*, and *Rgs3* in the case of tR > L (Figure 2D). In contrast, the tR > K p30 C/EBPα mutant induced a small number of pseudogenes (Figure 2D), which may potentially be linked to unknown regulatory mechanisms.^20^

In summary, the data show that alterations of the arginine side chain in positions R140, 147, and 154 have a significant impact on the transdifferentiation potential of p30 C/EBPα. In comparison to WT p30 C/EBPα, non-conservative mutations that result in the loss of charge (R to A, L substitutions) or mimic methylation (R to L) promote myeloid transdifferentiation, whereas conservative R to K mutations, which retain side chain charges but obliterate arginine specific methylation, constrained transdifferentiation.

### Phenotypic potential of p30 C/EBPα mutants in progenitor cells

In the absence of p42, p30 C/EBPα induces myeloid commitment and enhances leukemic expansion of progenitor cells, reflecting its functions as a regulator of cell fate and as a myeloid driver oncoprotein.^7^ We therefore examined the functional characteristics of WT and p30 C/EBPα mutant variants using serial colony replating of early progenitor cells derived from mouse bone marrow (lineage depleted, Sca1 positive, c-Kit negative, LSK; bone marrow cells derived from Cebpa^*fl/fl*^Cebpb^*fl/fl*^ “WT” mice).^21^^,^^22^ LSK cells were transduced with p30 C/EBPα or control vector and GFP^+^ sorted cells were plated in semi-solid medium supplemented with cytokines and replated following a standard regime to determine how p30 constructs affect the replication capacity and colony characteristics.^23^

**Figure 3:**
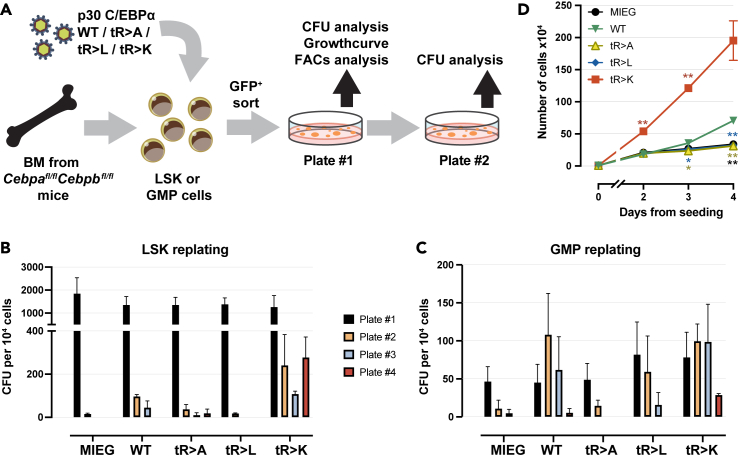
Serial replating potential of progenitor cells expressing WT or mutant p30 C/EBPα (A) Schematic representation of serial replating assay. Bone marrow cells were harvested from *Cebpa*^*fl/fl*^*Cebpb*^*fl/fl*^ mice. Cells were enriched for c-Kit using magnetic cell sorting, followed by LSK and GMP flow cytometry sorting. Retroviral infections were performed 24 h after cell harvesting (day 0). GFP^+^ cells were sorted at day 2-pi and plated. Colonies were counted/re-seeded every 7 days. (B and C) Colony formation recorded over four passages of replating LSK cells (B) and GMP cells (C). Colony numbers were determined by manual counting on scanned microscopic images. Colony formation rates per 10^4^ cells from three biological replicates are shown. Data are shown as mean ± SEM. (D) Growth curve of accumulative cell counts using cells derived from Plate #1 of total c-Kit enriched bone marrow replating (as shown in Figure S3A). Data are shown as mean ± SEM, significance was determined by two-way ANOVA analysis followed by Dunnett’s multiple comparisons test, as shown by asterisks in matching color, ∗*p* ≤ 0.05, ∗∗*p* ≤ 0.01, ∗∗∗*p* ≤ 0.005, ∗∗∗∗*p* ≤ 0.001. Results of one experiment with three technical replicates are shown. Three independent experiments were performed with similar results.

Figure 3A schematically shows the examination of the colony morphology, clonogenicity, and proliferation capacity. Serial replating assays of c-Kit-enriched cells showed that GFP^+^ cells expressing tR > K p30 C/EBPα maintained replating capacity up to or beyond the fourth passage, whereas all other p30 C/EBPα constructs led to loss of clonogenicity after the second and third rounds of replating (Figure S3A). We next compared the serial replating capacity of early progenitor LSK cells and GMP cells (defined as lineage^−^Sca-1^−^c-Kit^+^FcgRIII^+^, Figure S3B) expressing p30 C/EBPα mutants side-by-side. For both hematopoietic developmental stages, the tR > K p30 C/EBPα mutant exhibited similar replating capabilities up to the fourth passage (Figures 3B and 3C). However, the cells derived from the LSK displayed more colonies in comparison to GMPs, indicating the superior clonogenicity of very early progenitors.

Cells derived from the starting c-Kit-enriched culture (Plate #1, Figures 3A and S3A) were further examined by proliferation analysis, including cell counting, colorimetric (WST-1) proliferation assay, and dye dilution assay. Notably, the tR > K p30 C/EBPα mutant expressing cells exhibited the highest increase in cell number (Figure 3D) and proliferation (Figures S3C and S3D) across all three assays compared to the other p30 C/EBPα variants. The tR > K p30 C/EBPα mutant also exhibit faster cytoplasmic dye dilution (CellTrace Violet, Figure S3D), indicating acceleration of the cell cycle. The WT p30 C/EBPα construct also induced proliferation and clonogenicity, although to a lesser extent than that of tR > K. Collectively, the results suggest that among the p30 C/EBPα constructs tested, the tR > K p30 C/EBPα variant augments both the clonogenic potential and the proliferation of progenitor cells at the HSC and GMP stages.

**Figure 4:**
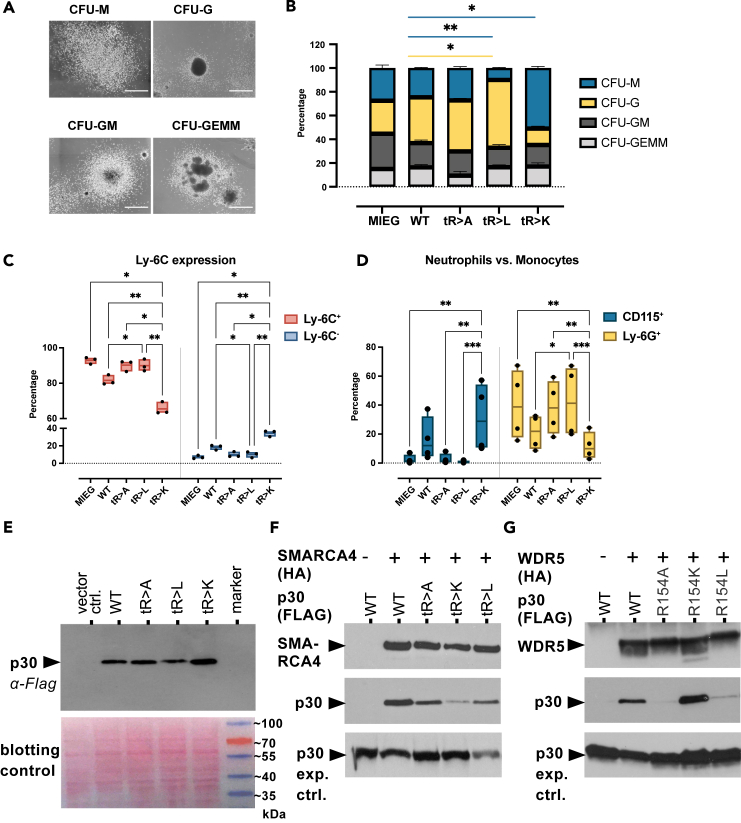
Myeloid differentiation induced by WT and mutant p30 C/EBPα (A) Characteristic types of colony morphologies on Plate #1. Scale bar 100 μm. Determination of colony types was as listed in Thermo Fisher Scientific (provider of methylcellulose based medium M3434 for colony formation assay used in this experiment). (B) Percentage of colony types on Plate #1. Constructs are indicated below stacked bar graphs. Colonies were counted using scanned images and categorized according to (A). Representative results from one experiment with three technical replicates are shown. Data are shown as mean ± SEM, significance was determined by two-way ANOVA analysis followed by Dunnett’s multiple comparisons test. Only significant comparison between WT and other constructs is shown, ∗*p* ≤ 0.05, ∗∗*p* ≤ 0.01, ∗∗∗*p* ≤ 0.005, ∗∗∗∗*p* ≤ 0.001, color code representing colony types are shown on the right. (C and D) Percentage of Ly6C^−^ and Ly6C^+^ population (C) and Ly6C^+^CD115^+^ monocytes and Ly6C^+^Ly6G^+^ neutrophils (D) on Plate #1. Data are shown as mean ± SEM, *n* = 3, significance was determined by two-way ANOVA analysis followed by Dunnett’s multiple comparisons test, ∗*p* ≤ 0.05, ∗∗*p* ≤ 0.01, ∗∗∗*p* ≤ 0.005, ∗∗∗∗*p* ≤ 0.001, only significant comparisons are shown. (E) Upper panel: Expression of WT and triple mutant p30 C/EBPα constructs in retroviral packaging plat-E cells. Protein lysates were separated by SDS-gel electrophoresis, blotted and probed with anti-FLAG to reveal p30 C/EBPα expression, as indicated. Lower panel: Ponceau staining as lysate and blotting control of the same blot. (F) Interaction of WT or triple mutant p30 C/EBPα with SMARCA4. HEK293T were co-transfected with SMARCA4-HA and C/EBPα-FLAG constructs, as indicated. SMARCA4 was immunoprecipitated and detected with anti-HA. Co-immunoprecipitated WT or triple mutant p30 C/EBPα was detected by anti-FLAG. Underneath: Expression controls of p30 C/EBPα constructs in cell lysates. (G) Interaction of WT or single mutant p30 C/EBPα with WDR5. Co-immunoprecipitation protocol as in F, from cells co-transfected with WDR5-HA and C/EBPα-FLAG constructs, as indicated. Underneath: Expression controls of p30 C/EBPα constructs in cell lysates.

Next, we examined the fates of cells derived from LSK colonies (Plate #1, Figures 3A and S3A) that expressed WT or mutant p30 C/EBPα. Four morphological categories were used to determine colony types (Figure 4A). The predominant type of colonies induced by p30 C/EBPα tR > L exhibited a typical granulocytic CFU-G morphology, characterized by a compact core surrounded by dispersed small cells (Figure 4B). In contrast, most colonies generated by tR > K p30 C/EBPα resembled monocytic CFU-M, with highly dispersed colonies, containing large cells (Figure 4B). Thus, the emergence of CFU-G and CFU-M type colonies correlated with the tR > L and tR > K versions of p30 C/EBPα, respectively. Cytology of cells, as revealed by cytospins obtained from pooled colony populations, further supported these observations, showing a high proportion of typical neutrophil granulocytes at different maturation stages (bi- or multilobed, polymorphic nuclei, cytoplasmic azurophilic granules) in tR > L p30 C/EBPα cells while the majority of tR > K p30 C/EBPα were monocytes (rounded shape, pale blue-gray cytoplasm, small lilac granules, and vacuoles) (Figure S4A).

Cells from colony Plate #1 were also analyzed by flow cytometry to distinguish the distribution of granulocytes (Ly6C^+^Ly6G^+^CD115^-^) and classical monocytes (Ly6C^+^Ly6G^−^CD115^+^) (Figure S4B). We noticed an accumulation of Ly6C^−^ cells in colonies with tR > K p30 C/EBPα, while these cells constituted less than 20% in the other groups (Figure 4C). Within Ly6C^+^ population, differentiation toward Ly6G^+^ granulocytes was pronounced with tR > L p30 C/EBPα, while CD115^+^ monocyte differentiation was favored with tR > K p30 C/EBPα (Figure 4D). The phenotypes induced by WT p30 C/EBPα fell between those induced by tR > A or tR > L p30 C/EBPα on one end of the spectrum and tR > K p30 C/EBPα on the other. Notably, the accumulated Ly6C^−^ population in tR > K p30 C/EBPα cells exhibited no overlap with other myeloid marker expressing populations (Ly6G, CD115, Cd11b, F4/80), ruling out the possibility they represent non-classical Ly6C^lo/neg^ monocytes, instead they likely represented an undifferentiated population (Figure S4C).

Finally, we examined whether the undifferentiated Ly6C^−^ population accounted for the enhanced proliferation of tR > K p30 C/EBPα expression cells using a dye dilution assay. Staining of Ly6C was integrated in the assay shown in Figure S4D. The dye dilution rate of Ly6C^+^ and Ly6C^−^ subsets showed minimal differences between tR > A, tR > L p30 C/EBPα expressing cells, while tR > K and WT p30 C/EBPα expressing cells showed higher division rate in the Ly6C^+^ subset (Figure S4D). In both Ly6C subsets, proliferation was highest with tR > K (Figure S4D). During a 7-day time course, we observed an overall trend in that the Ly6C^+^ population gradually diminished (in population percentage and cell fitness, microscopic observation, data not shown), while the Ly6C^−^ cells maintained growth. This implies that beside cell cycle progression, prevention of cell death may also play a role in tR > K p30 C/EBPα mutant cells. In these experiments (Figures 3 and 4), the endogenous *Cebpa*,*b* genes were not deleted, as the procedures for gene deletion would have opposed the maintenance of the primary progenitor status. Accordingly, the *Cebpa*^*fl/fl*^*Cebpb*^*fl/fl*^ cells are analogous to wild-type cells. Nevertheless, cell proliferation and differentiation of transgene-expressing cells were significantly different from the vector control group and resembled the differentiation observed with dKO-B cells (Figure 1). This suggests that the retrovirally expressed transgenes were dominant over any effects that might have been elicited by the endogenous *Cebpa*,*b* genes, as assessed by colony formation and differentiation of bone marrow cells. Altogether the data suggest that the tR > K p30 C/EBPα mutant supports proliferation of hematopoietic precursors and myeloid progenitors at various differentiation stages.

Seminal genetic studies conducted in *Drosophila melanogaster* and *Saccharomyces cerevisiae* have unveiled numerous components of the Trithorax/COMPASS-like/MLL and SWI/SNF/BAF complexes. These complexes, initially identified for their roles in segmental transformation and mating-type switching, have since been recognized as multi-component protein complexes involved in chromatin modification and remodeling, respectively. Both complexes are categorized within the Trithorax-group, a set of evolutionarily highly conserved epigenetic regulators crucial for cell type specification and tumorigenesis, including hematopoiesis and leukemogenesis.^24^^,^^25^

Previous studies have demonstrated physical and functional interactions of both complexes with C/EBPα during critical developmental processes in hematopoietic differentiation and proliferation.^9^^,^^26^^,^^27^ In an endeavor to integrate our biological findings with previously reported interactions, we investigated the core components of SWI/SNF and MLL complexes for their interaction with WT and mutant p30 C/EBPα using co-immunoprecipitation assays (Figures 4E–4G).

The interaction between SWI/SNF and C/EBPα has been previously documented to rely on the N-terminus of p30 C/EBPα.^28^^,^^29^^,^^30^ Additionally, proteomic analyses have shown an enrichment of SWI/SNF with the tR > L p30 C/EBPα mutant and methylated R140 over the WT, including the core ATPase subunit SMARCA4 (Brm).^12^ As illustrated in Figures 4E and 4F, co-immunoprecipitation confirmed that both differentiation enhancing tR > L, tR > A p30 C/EBPα mutants exhibited a stronger interaction with SMARCA4 (Brm) compared to the proliferation-enhancing tR > K p30 C/EBPα mutant, which displayed a reduced interaction.

Progenitor proliferation induced by p30 C/EBPα relies on the recruitment of the MLL complex through the adapter protein WDR5, which is also a constituent of several other chromatin regulatory complexes.^9^^,^^26^^,^^27^^,^^31^^,^^32^^,^^33^ To investigate the interaction of WDR5 with differentially methylated p30 C/EBPα N-termini, we employed the motif-based screening method of immobilized tiled peptides (PRISMA; utilizing both WT and post-translational modification-modified tiled peptide configurations).^14^ As depicted in Figures 4G and S4E–S4H, WDR5 exhibited highly specific binding to peptides containing the unmethylated R154 side chain configuration or to positive control peptides derived from histone H3 or MLL.^31^^,^^34^ Symmetric or asymmetric methylation at position R154 of p30 C/EBPα peptides, or substitution with alanine or citrulline, hindered the binding of WDR5, thus confirming the selectivity and methylation sensitivity of WDR5 interactions. Finally, co-immunoprecipitation assays validated the association of WDR5 with both WT and R154K p30 C/EBPα in cells, whereas the R154A and R154L p30 C/EBPα mutants exhibited reduced association with WDR5 (Figure 4G).

In summary, these findings highlight how distinct arginine residues and their modification status in p30 C/EBPα differentially influence the interaction with critical components of the MLL and SWI/SNF complexes.

## Discussion

Contrary to the prevailing concept that p30 C/EBPα primarily functions as a dominant-negative variant of the full-length p42 C/EBPα, our data show that p30 C/EBPα instead can direct cell fate and progenitor proliferation in a PTM-dependent fashion. Three conserved arginine residues (R140, 147, 154) located in the N-terminus of p30 C/EBPα are critically involved and point mutations at these sites distinctly modify the biological functions of p30 C/EBPα in lineage commitment, differentiation, and proliferation. Our data add evidence to the concept that arginine side chain methylation of pioneering TF can determine epigenetic outcomes upstream of histone modifications.^15^^,^^35^^,^^36^

The most plausible explanation for our data is that the p30 C/EBPα mutants represent hypo- and hypermorphic mimics of arginine methylation. Accordingly, tR > K p30 C/EBPα represents the unmethylated state of WT p30 C/EBPα and facilitates progenitor expansion and monocytic cell fate, while methylation of arginine side chains, represented by tR > L p30 C/EBPα, is associated with restrained progenitor expansion and granulocyte differentiation. This interpretation aligns with previous biochemical data demonstrating that the arginine methylation status of C/EBPs profoundly alters their interactomes and biological functions.^12^^,^^14^^,^^17^^,^^37^ Hence, forthcoming studies decoding the upstream and downstream regulatory p30 C/EBPα network in hematopoietic progenitor biology and leukemia should consider the cellular machinery involved in C/EBPα arginine methylation.

The tR > A,L p30 C/EBPα mutants and the tR > K evoke contrasting phenotypes in terms of cell lineage commitment. The tR > A,L mutants promote granulopoietic differentiation and attenuate clonogenicity and proliferation. In contrast, the tR > K p30 C/EBPα mutant, that retains the positively charged side chains, supports proliferation and elicits a monocytic phenotype.

At the transcriptional level, we observed that both tR > A and tR > L induced the expression of lineage-specific transcription factors *Gfi1* (associated with the granulocytic lineage) and *Spi1* (encoding PU.1, associated with the monocytic lineage), consistent with previous findings involving p42 C/EBPα. However, the expression levels of these genes were compared only among p30 C/EBPα constructs and not with p42 C/EBPα. Therefore, it remains unclear how p30 and p42 compare in terms of the magnitude of PU.1 and GFI1 expression and their combinatory roles in driving monocytic versus granulocytic lineage commitment.^38^^,^^39^

Furthermore, Ly6G + neutrophils were absent in transdifferentiation experiments using Cebpa−/−Cebpb−/− B cells, whereas they were abundantly present in lineage differentiation experiments involving Cebpafl/flCebpbfl/fl bone marrow-derived cells. Accordingly, our data suggest that the tR > A and tR > L variants of p30 C/EBPα retain some functions similar to those of p42 C/EBPα, but not its full potential and that endogenous C/EBPα and C/EBPβ play a role in neutrophil differentiation. Additionally, this implies that the synergy and cross-regulation between p42 and p30 C/EBPα, as well as between C/EBPα and C/EBPβ, in conjunction with GFI1 and PU.1, need to be considered in future studies to fully understand lineage commitment.

The WT p30 C/EBPα settles between both phenotypes, in consistency with its arginine side chains in either unmodified or (partially) modified configuration. In accordance, methylation of human R142 (equivalent to rat R140) and the tR > L mutant were previously shown to enhance interaction with SWI/SNF, whereas methylation of R154 inhibits binding of WDR5 that mediates binding of the MLL complex.^12^^,^^27^^,^^32^ Thus, the differential interactions with SWI/SNF and MLL comply with the state of arginine modifications and with the biology of the p30 C/EBPα mutants, suggesting an important mechanistic connection.

Interestingly, the p30 C/EBPα triple mutants amplify the phenotypes that were already observed with single-site mutants (see Figure 2), suggesting individual and combinatorial functions of the adjacent arginine side chains. How can the functional co-operativity of adjacent arginine PTMs with their individual specificity be reconciled? PTM-dependent graduated non-stoichiometric functions are common biochemical features of linear regulatory protein motifs, including SLiMs.^40^^,^^41^^,^^42^ In the case of C/EBPα, we envisage that differential arginine modifications may “titrate” the reorganization propensity e.g., by structural “breathing” of the p30 C/EBPα N-terminus to modulate the access and function of its multivalent TE3-PPI motifs and, consequently, enable alternative biological outcomes. Considering the recent observation that C/EBPα undergoes biomolecular condensation that depends on aromatic amino acids in the intrinsically disordered N-terminal region, one may also consider phase-separation processes to be involved that are sensitive to arginine methylation.^43^ Notably, three highly conserved tyrosine residues (Y129/136/145) are located in the vicinity of the triple arginine residues in p30 C/EBPα. Alternating R-Y residues are a typical feature of IDRs that engage in non-covalent π-cation side chain interactions during polypeptide coalescence that are disrupted by arginine side chain methylation. Although speculative, this may explain why both, leucine and alanine substitutions of R140/147/154 p30 C/EBPα display similar biological outcomes, as neither alanine nor leucine side chains engage in pi-cation based interactions, while replacement with lysine can retain this function.^44^^,^^45^^,^^46^^,^^47^

### Limitations of the study

The findings presented in this study provide insights into key functions of the truncated p30 C/EBPα isoform and may hold promise for future targeted pharmacological interventions in p30 C/EBPα driven leukemia. However, the observations also raise questions about the dynamics and the enzymatic machinery involved in p30 C/EBPα methylation, its connections to cytokines and signaling pathways, intracellular transmission cascades, the underlying molecular genetics of chromatin structure, gene regulation, and homeostatic or leukemic effects in the living organism. As our studies are currently limited to *in vitro* assays, important future tasks will have to address C/EBP modifications in relation to normal hematopoiesis and disease in the living organism. Furthermore, investigating the impact of the respective p30 C/EBPα arginine PTMs in the p42 C/EBPα context will be important. Further, it will also be important to consider the positional isomeric status and the degree of arginine modifications, i.e., mono-, symmetric, and asymmetric arginine di-methylation, in relation to upstream signals and downstream effects, in analogy to what has been termed “histone code” in chromatin regulation. Moreover, methylation of arginine side chains appears to be largely irreversible, suggesting that it is a regulatory endpoint, except that citrullination by peptidyl arginine deiminases (PADI) may remove methylated and unmethylated arginine side chains to create a new side chain configuration.^48^

### Conclusions

In summary, the results shown here serve as a foundation and starting point, more comprehensive research on the connections between p30 C/EBPα PTM in the intact organism and its biological impact will be required. While the current study falls short of a holistic analysis, our data provide a rational of the dichotomous function of p30 C/EBPα in progenitor expansion and differentiation. This study may encourage further investigations of the physiological placement and impact of p30 C/EBPα PTM and its turnover in normal hematopoiesis and disease and as a pharmacological target.

## Resources availability

### Lead contact

Further information and requests for resources and reagents should be directed to and will be fulfilled by the lead contact, Achim Leutz, aleutz@mdc-berlin.de.

### Materials availability

This study did not generate new unique reagents.

### Data and code availability

• The RNA sequencing data are publicly available at the National Center for Biotechnology Information with Gene Expression Omnibus accession no. GEO: GSE266544.
• This paper does not report original code.
• Any additional information required to reanalyze the data reported i this paper is available from the lead contact upon request.

## Acknowledgments

We thank Hans-Peter Rahn and Kirstin Rautenberg (MDC, Berlin, Germany) for the flow cytometry support; Alexandra Patmanidi (MDC, Berlin, Germany) and Vladimír Beneš (Genomics Core Facility, EMBL, Heidelberg, Germany) for the RNA-seq support; the team of the Protein Production & Characterization Technology Platform at the MDC (https://www.mdc-berlin.de/ppcp) for the technical assistance.

## Author contributions

L.T.N.: investigation, formal analysis, original draft, visualization, validation; K.Z.: investigation, formal analysis, data curation, validation; E.K.-L.: investigation, formal analysis, methodology, resources, validation, supervision; D.D.: formal analysis, methodology; A.S.: investigation, formal analysis; J.S.: investigation, formal analysis; A.M.: investigation, methodology, supervision; A.L.: conceptualization, supervision, project administration, funding acquisition, methodology, investigation, review and editing.

## Declaration of interests

The authors declare no competing interests.

## STAR★Methods

### Key resources table

**Table:** 

REAGENT or RESOURCE	SOURCE	IDENTIFIER
**Antibodies**		

CD115 APC	eBioscience	#17-1152-80
CD11 b PE	BD Pharmingen	#557397
CD19 PE-Cy7	eBioscience	#25-0193-81
Ly6G BV421	BioLegend	#127628
Biotin-conjugated cKit antibody	BioLegend	#105803
Anti-biotin magnetic beads	Miltenyi Biotec	#130-090-485
CD117 (cKit) PE-Cy7	eBioscience	#25-1171-82
Streptavidin PECy-7	BioLegend	#405206
Ly6A/E (Sca-1) PE	BD Pharmingen	#553336
CD3e APC (Lineage cocktail)	BD Pharmingen	#553066
Ly6C/Ly6G APC (Lineage cocktail)	BD Pharmingen	#553129
CD11b APC (Lineage cocktail)	BD Pharmingen	#553312
Ter119 APC (Lineage cocktail)	BioLegend	#116211
B220 APC (Lineage cocktail)	BD Pharmingen	#553092
CD11c APC (Lineage cocktail)	BD Pharmingen	#550261
CD5 APC (Lineage cocktail)	eBioscience	#17-0051-82
CD115 APC (Lineage cocktail)	BioLegend	#135509
CD16/32 PE	BD Pharmingen	#561727
CD117 (cKit) PE-Cy7	eBioscience	#25-1171-82
CD34 Alexa Fluor 647	BioLegend	#152205
Ly-6G Alexa Fluor 700	BioLegend	#127621
Ly-6C APC-Cy7	BioLegend	#128025
F4/80 Pacific Blue	BioLegend	#123123
CD115 Brilliant Violet 605	BioLegend	#135517
CD11b Brilliant Violet 711	BioLegend	#101241
CD11c PerCP-Cy5.5	BioLegend	#117327
anti-WDR5	Abcam	#ab56919
anti-mouse HRP	Invitrogen	#31431

**Bacterial and virus strains**		

E. coli T7 Express cells	NEB	#C2566H
Platinum-E Retroviral Packaging Cell Lines	N/A	N/A

**Chemicals, peptides, and recombinant proteins**		

Polybrene	Sigma	#TR-1003-G
Polyethylenimine	Polysciences	#24765-2
Red blood cell lysis solution	Miltenyi	#130-094-183
Methocult MC3434	STEMCELL Technology	#03444
WST-1 reagent	Roche	#05015944001
HisTrap FF crude column	Cytiva	#17528601
Superdex 200 prep grade column	XK 26 × 60, Cytiva	# 28-9893-36
RNAeasy Kit	Qiagen	#74104
mIL-3	STEMCELL Technology	#78042.1
mIL-6	STEMCELL Technology	#78052.2
mSCF	STEMCELL Technology	#78064.1

**Critical commercial assays**		

QuikChange Site-Directed Mutagenesis Kit	Stratagene	#200518
CellTrace Violet	ThermoFisher Scientific	#C34557
NextSeq500 platform	Illumina, EMBL Genomics Core Facility	N/A

**Deposited data**		

Raw and analyzed RNAseq data	This study	GEO: GSE266544

**Experimental models: Cell lines**		

v-Abl transformed Cebpa^Δ/Δ^Cebpb^Δ/Δ^ pre-B cells	Cirovic et al., 2017^18^	N/A
Bone marrow derived Cebpa^fl/fl^Cebpb^fl/fl^ cells	This study	N/A
HEKT-293 cells	This study	N/A

**Oligonucleotides**		

See Table S1	This study	N/A

**Recombinant DNA**		

pMSCV-IRES-EGFP (MIEG) retroviral vectors	Cirovic et al. 2017^18^	N/A
pRARE2 plasmid	Novagen	N/A

**Software and algorithms**		

Flowjo^TM^	FlowJo, LLC	V8
EVOS^TM^ FL^TM^ auto imaging system	Thermo Fisher Scientific	N/A

### Experimental model and study participant details

Experiments involved bone marrow-derived cells were extracted from *Cebpa*^*fl/fl*^*Cebpb*^*fl/fl*^ mice of both sexes, aged 8–12 weeks. The *Cebpa*^*fl/fl*^*Cebpb*^*fl/fl*^ mice were maintained and handled in compliance with the German Animal Welfare Act, following approvals by the local ethics committee Berlin State Office for Health and Social Affairs (LaGeSo).

### Method details

#### Cell culture

The LMT system employed the Cebpa^Δ/Δ^Cebpb^Δ/Δ^ v-Abl transformed pre-B cell line generated as described before.^18^ Briefly, bone marrow cells were isolated from the femur and tibia of 8- to 9-week-old *Cebpal*^*fl/fl*^-crossed (Zhang et al., 2004) and *Cebpb*^*flllf*^-crossed (Sterneck et al., 2006) *Cebpa*^*fl/fl*^*;Cebpb*^*fl/fl*^ mice and C57BL/6J wild-type controls. After erythrolysis, bone marrow cells were transduced with v-Abl-expressing retroviral supernatants and 8 μg/mL hexadimethrine bromide in complete DMEM (10% fetal calf serum (FCS), 10 mM HEPES, and penicillin/streptomycin (Gibco)) supplemented with 50 μM β-mercaptoethanol. Bone marrow cells were washed on the following day and medium was changed every other day. A stable cell line with a pre-B cell-like phenotype (CD19^+^, c-kit−, CD25^+^, IgM−) emerged after 4 weeks of culture. For *loxP* site recombination, 5 × 10^5^ B cells were washed three times in serum-free DMEM and incubated with purified TAT-Cre protein (50 μg/mL; a gift from Dr. K. Rajewsky) in serum-free DMEM at 37°C for 45 min. The cells were then washed and cultured for 24 h and seeded subsequently as single-cell clones (by fluorescence-activated cell sorting (FACS)) into 96-well plates. Deletion of the *Cebpa* and *Cebpb* genes was checked by PCR to identify double-knockout clones. The established clone 18 of this cell line was used throughout this study and termed dKO-B cells. dKO-B cells were maintained in complete DMEM (10% fetal bovine serum (FBS), 10 mM HEPES and penicillin/streptomycin) supplemented with 50 mM β-mercaptoethanol. For retrovirus generation, Plat-E was used as packaging cell line.

Bone marrow-derived progenitors and stem cells were culture in IMDM supplemented with 10 ng/mL IL-3, 10 ng/mL IL-6 and 20 ng/mL mSCF (termed cytokine supplemented-IMDM).

#### Expression constructs and retroviral infection

C/EBP genes were expressed using pMSCV-IRES-EGFP (MIEG) retroviral vectors as described.^18^ Point mutations in *Cebpa p42* were introduced by site-directed mutagenesis using the QuikChange Site-Directed Mutagenesis Kit (Stratagene #200518). Primers used for single mutant exchange were listed in key resources table. Mutated C/EBPa p42 constructs were used as templates for synthesizing p30 C/EBPα constructs using polymerase chain reactions and EcoRI/XhoI restriction site-targeted primers as listed. p30 C/EBPα fragments were cloned into MIEG to generate pMSCV_p30-mutant_3xFLAG_IRES_EGFP vectors. Triple mutants tR > A, tR > L, tR > K were commercially synthesized (GenScript) and cloned into MIEG.

Production of infectious retroviral supernatant were carried out using polyethylenimine to transfect 5 μg of retroviral plasmids into Plat-E packaging cells. Supernatant was collected at 48 h and 72 h after transfection. Retroviral supernatant containing various C/EBP constructs was added to dKO-B cells together with 8 μg/mL polybrene (Sigma). Cells were infected by spinoculation at 2000 rpm at 37°C for 1 h, following by overnight incubation. Cells were then washed and resuspended in fresh medium. Transdifferentiation of dKO-B cells was executed as described.^18^

#### Cell transfection

HEKT-293 cells were transfected with C/EBPα WT and mutant expression vectors in the absence or presence of hWDR-HA or hBrm-HA (SMARCA4) as indicated, using Polyethylenimine according to the manufacturer’s instructions (PEI, Polysciences #24765-2).

#### Flow cytometric analysis and sorting

After harvesting at indicated time points, cells were washed in cold FACS buffer (2% FCS, 2 mM EDTA in PBS) and incubated with Fc-blocking reagent (1:200, TruStain FcX, anti-mouse CD16/32, BioLegend). After washing, cells were proceeded to labeling with fluorescence-conjugated antibodies at 4°C for 30 min in the dark. Stained cells were washed and resuspended in FACS buffer containing propidium iodide (PI, BD Biosciences) for live-dead discrimination. Samples were measured using LSRFortessa analyzer. Cells were sorted using FACSAria II/III instruments (BD Biosciences). Gentamicin was added to buffer and post-sort cell culture medium at final concentration 10 μg/mL. Sorted cells were spun down in gentle cycle (700 rpm, 7 min) and resuspended in fresh medium. Measurement was recorded using FACSDiva and analyzed using Flowjo software v8.

#### Isolation of bone marrow cells and LSK cells

Bone marrow-derived cells were isolated from femur, tibia and part of hip joints of *Cebpa*^*fl/fl*^*Cebpb*^*fl/fl*^ mice. Bones were flushed with cold PBS using a syringe and a 24-gauge needle (for serial replating of total cKit-enriched bone marrow cells) or crushed in cold PBS (for serial replating of LSK and GMP cells) under sterile condition. Cell suspension was filtered through a 70 μM cell strainer before incubation with red blood cell lysis solution (Miltenyi) for 8–10 min on ice. Cells were washed and cultured in cytokine supplemented-IMDM or resuspended in PBS for further processing.

#### Enrichment of cKit^+^ cells

Isolated bone marrow cells were incubated with biotin-conjugated cKit antibody for 20 min at 4°C. After washing, anti-biotin magnetic beads were added to cell suspension (20 μL beads per 10^7^ cells) and incubated for 30 min at 4°C. Magnetically labeled cells were washed and resuspended in max. 10^9^ cells/mL and passed through equilibrated selecting columns (LS Column, Miltenyi) mounted on a MidiMACS or a QuadroMACS separator. Cell-loaded columns were washed 3 times with MACS buffer, transferred to a 15 mL centrifuge tube and eluted with 1 mL MACS buffer. Cells were then washed and cultured in cytokine supplemented-IMDM or prepared for further processing.

#### Colony serial replating assay

Colony serial replating assay was performed on C/EBPα p30 (WT or mutants) infected, GFP^+^ sorted bone marrow cells. Directly after sorting, cells were resuspended in cytokine supplemented-IMDM at 5 × 10^4^ cells/mL. Cell mixture was diluted in Methocult medium (MC3434, STEMCELL Technology) to obtain a final concentration of 5000 cells/mL. Semi-solid MC3434 cell mixtures were plated at 1 mL mixture/well (supplemented with 10 ng/mL IL-3, 10 ng/mL IL-6 and 20 ng/mL mSCF) in a 6-well meniscus-free dish (SmartDishTM, STEMCELL Technology) using a 3cc syringe and a blunt-end 16- gauge needle (STEMCELL Technology). Colony dishes were scanned using EVOS FL auto imaging system (Thermo Fisher Scientific) every 7 days for 4 passages (day 7, day 14, day 21 and day 28) at 4× magnification. Colonies with ≥50 cells were counted and classified by microscopical inspection based on morphology. Phenotyping of colonies was based on colony size, shape and compactness, cell size and shape, cell distribution within and around colonies.

To replate, cell suspension in three replicates were pooled, spun and washed with PBS. Colonies were well-suspended to obtain single cells suspension and diluted to 5 × 10^4^ cells/mL in cytokine supplemented-IMDM. Cell suspension was added to MC3434 aliquots and seeded as described above. Leftover cells of day 7 plates (Plate 1) were subjected to further analysis, including flow cytometric analysis, proliferation analysis and cytospin.

#### Proliferation assays

Growth curves were determined by cell counting and WST-1 assay. For cell counting, 10^5^ cells were seeded in 1mL cytokine supplemented IMDM into a 24-well plate in triplication. Cells were count every 24 h by staining with Trypan blue and using a Neubauer chamber.

For WST-1 assay, 10^4^ cells were seeded in 100 μL cytokine supplemented IMDM into a 96-well plate in triplication. Every 24 h, 10 μL of WST-1 reagent (Roche) was added to each well (1:10 dilution) and incubated at 37°C. After 60 min, absorbance of 450 nm wavelength was measured using an iMark microplate absorbance reader (Bio-Rad). A blank control wells, which contained equivalent cell culture medium was used for normalization of OD values. Proliferation rate was determined by dye-dilution assay using CellTrace Violet (ThermoFisher Scientific). Cells were processed following manufacturer’s suggested procedure. An aliquot of the culture suspension was drawn at indicated time points and labeled with antibodies including Ly6C, Ly6G, CD115 and CD11b. Labeled cells were measured as described; PI was used for live-dead determination.

#### May-Grünwald/Giemsa staining

Cells were spun onto glass slides using an Aerospray Slide Stainer cytocentrifuge (Wescor) at 500 rpm for 5 min. Air-dried slides were fixed with methanol before immersed into May-Grünwald solution for 5 min. After washing, slides were immersed in freshly prepared Giemsa solution for 35–45 min. Stained slides were rinsed, air-dried, and mounted with mounting solution (Roti-Histokitt II). Stained cells were observed under the microscope and captured at multicolor mode (EVOS, Invitrogen).

#### Protein extraction, immunoprecipitation and western blot

Production of whole cell lysates and immunoprecipitation of WT or mutant C/EBPα proteins were performed as previously described.^37^ Briefly, cells were lyzed for 30 min on ice (20 mM HEPES pH 7.8, 150 mM NaCl, 1 mM EDTA pH 8, 10 mM MgCl_2_, 0.1% Triton X-100, 10% glycerol, protease inhibitor cocktail (Merck), 1 mM DTT, 1 mM PEFA bloc (Böhringer), briefly sonicated and lysates were cleared by centrifugation. Immunoprecipitation was performed with antibodies as indicated for 2 h at 4°C. Immunoprecipitated proteins were collected on Protein-G Dynabeads (Invitrogen #10004D), separated by SDS-PAGE (Mini PROTEAN TGX, 4–15%, Bio-Rad #5671084) and immunoblots were incubated with antibodies (HA, Covance #MMS-101R; Flag, Sigma #F3165), as indicated and visualized by ECL (GE Healthcare, UK).

#### WDR5 protein production

DNA encoding amino acids 24–334 of human WDR5 was subcloned into the pQLinkH vector. The N-terminal His_7_-tagged protein was produced at 17°C using *E. coli* T7 Express cells (NEB) bearing the pRARE2 plasmid (Novagen). The purification protocol comprised mechanical cell lysis by sonication (SONOPULS HD 2200, Bandelin), affinity chromatography on a 5 mL HisTrap FF crude column (Cytiva) and size-exclusion chromatography on a Superdex 200 prep grade column (XK 26 × 60, Cytiva) pre-equilibrated with 20 mM HEPES-NaOH pH 7.5 and 0.5 M NaCl. The purified protein was supplemented with 1 mM DTT and 2.5% (v/v) 1,2-propanediol, flash-frozen with liquid nitrogen and stored at −80°C until usage.^49^

#### WDR5 interaction mapping on spot-synthesized tiling p30 C/EBPα peptides

To map post-translational dependent WDR5 interaction with the three arginine residues R140, R147, R154 contained in the p30 C/EBPα N-terminus a protein interaction screen on a custom PepSpot cellulose membrane (PRISMA; JPT Peptid Technologies, Berlin)^14^ was performed using tiling peptides of 13 aa length spanning the critical Arginine residues (see also Figure S4). Arginine residues within peptides were modified to Rme2as, Rme2sym, citrulline or alanine. Binding specificity was controlled by histone H3 or Mlll1 peptides as indicated. The membrane was washed in binding buffer (20 mM HEPES pH 7.8, 100 mM KCL, 0.2 mM EDTA pH 8, 1.5 mM MgCl2, 20% glycerol, 1 mM DTT, 100 μg/mL tRNA) for 20 min. Bacterially expressed and purified N-terminally His7-tagged human WDR5 protein (aa24-334; 10 μg/mL) was incubated in binding buffer with the membrane on a rocking platform on ice for 30 min.^12^^,^^14^ Filters were washed 3 × 5 min with binding buffer and subsequently blocked for 20 min with Rotiblock (Roth #A151.1) at RT. The WDR5 specific antibody (Abcam) was incubated in Rotiblock for 20 min at RT. The membrane was washed twice for 5 min with TBST 0.01% Tween and incubated with anti-mouse HRP antibody (Invitrogen) for 15 min at RT. After washing twice with TBST 0.01% Tween for 5 min, the filter was developed with ECL (Amersham) and signals determined and quantified on a Licor scanner (C-DiGit Blot Scanner).

#### Bulk RNA-sequencing

After virus infection (as in Figure 1), EGFP^+^ cells were sorted at day 4 p.i. and subjected to bulk RNA-sequencing. Cell pellets were resuspended in RNA lysis buffer (RNeasy Kit, Qiagen) and stored at −80°C. Quadruplicate samples harvested in different batches were processed altogether using RNAeasy Kit (Qiagen). For RNA-seq, 1 μg of total RNA was used. The concentration of extracted total RNA was measured using the Qubit 3 Fluorometer (Thermo Fisher Scientific). Quality and integrity of RNA were measured using the Eukaryote Total RNA Nano assay on a Bioanalyzer 2100 (Agilent Technologies). Preparation of barcoded mRNA-seq library and sequencing using NextSeq500 platform (Illumina) with paired-end reading at 75 bps read-length were performed at the EMBL Genomics Core Facility (Heidelberg, Germany).

#### Bioinformatic analysis of mRNA sequencing data

Raw sequencing reads were aligned to the mm10 reference genome using STAR version 2.7.9a. Subsequently, htseq-count version 0.1 was applied for gene expression quantification. Normalization and differential gene expression were performed with DESeq2 using an adjusted *p*-value cutoff of 0.05 and an absolute fold change of 2 as parameters defining differential expression. Functional analysis was done with gProfiler using GO:BP and KEGG as an annotation resource.

### Quantification and statistical analysis

Data were analyzed for statistical significance by tests indicated at each figure using GraphPad Prism 10.1.1. For comparisons among the groups, ANOVA analysis with Dunnett’s multiple comparisons tests were used. Data are shown as mean ± standard error of the mean (SEM), significance are represented as ∗*p* ≤ 0.05, ∗∗*p* ≤ 0.01, ∗∗∗*p* ≤ 0.005, ∗∗∗∗*p* ≤ 0.001; ns, not significant.

## References

[1] Heath V., Suh H.C., Holman M., Renn K., Gooya J.M., Parkin S., Klarmann K.D., Ortiz M., Johnson P., Keller J. (2004). C/EBPalpha deficiency results in hyperproliferation of hematopoietic progenitor cells and disrupts macrophage development in vitro and in vivo. Blood.

[2] Pabst T., Mueller B.U., Zhang P., Radomska H.S., Narravula S., Schnittger S., Behre G., Hiddemann W., Tenen D.G. (2001). Dominant-negative mutations of CEBPA, encoding CCAAT/enhancer binding protein-alpha (C/EBPalpha), in acute myeloid leukemia. Nat. Genet..

[3] Skokowa J., Lan D., Thakur B.K., Wang F., Gupta K., Cario G., Brechlin A.M., Schambach A., Hinrichsen L., Meyer G. (2009). NAMPT is essential for the G-CSF-induced myeloid differentiation via a NAD(+)-sirtuin-1-dependent pathway. Nat. Med..

[4] Zhang P., Iwasaki-Arai J., Iwasaki H., Fenyus M.L., Dayaram T., Owens B.M., Shigematsu H., Levantini E., Huettner C.S., Lekstrom-Himes J.A. (2004). Enhancement of hematopoietic stem cell repopulating capacity and self-renewal in the absence of the transcription factor C/EBP alpha. Immunity.

[5] Calkhoven C.F., Bouwman P.R., Snippe L., Ab G. (1994). Translation start site multiplicity of the CCAAT/enhancer binding protein alpha mRNA is dictated by a small 5' open reading frame. Nucleic Acids Res..

[6] Nerlov C. (2004). C/EBPalpha mutations in acute myeloid leukaemias. Nat. Rev. Cancer.

[7] Kirstetter P., Schuster M.B., Bereshchenko O., Moore S., Dvinge H., Kurz E., Theilgaard-Mönch K., Månsson R., Pedersen T.Å., Pabst T. (2008). Modeling of C/EBPalpha mutant acute myeloid leukemia reveals a common expression signature of committed myeloid leukemia-initiating cells. Cancer Cell.

[8] Hayashi Y., Hirai H., Yao H., Yoshioka S., Satake S., Kamio N., Miura Y., Ashihara E., Fujiyama Y., Tenen D.G., Maekawa T. (2011). BCR/ABL-Mediated Myeloid Expansion Is Promoted by C/EBPβ, a Regulator of Emergency Granulopoiesis. Blood.

[9] Wesolowski R., Kowenz-Leutz E., Zimmermann K., Dörr D., Hofstätter M., Slany R.K., Mildner A., Leutz A. (2021). Myeloid transformation by MLL-ENL depends strictly on C/EBP. Life Sci. Alliance.

[10] Bereshchenko O., Mancini E., Moore S., Bilbao D., Månsson R., Luc S., Grover A., Jacobsen S.E.W., Bryder D., Nerlov C. (2009). Hematopoietic stem cell expansion precedes the generation of committed myeloid leukemia-initiating cells in C/EBPalpha mutant AML. Cancer Cell.

[11] Jakobsen J.S., Laursen L.G., Schuster M.B., Pundhir S., Schoof E., Ge Y., d’Altri T., Vitting-Seerup K., Rapin N., Gentil C. (2019). Mutant CEBPA directly drives the expression of the targetable tumor-promoting factor CD73 in AML. Sci. Adv..

[12] Ramberger E., Sapozhnikova V., Kowenz-Leutz E., Zimmermann K., Nicot N., Nazarov P.V., Perez-Hernandez D., Reimer U., Mertins P., Dittmar G., Leutz A. (2021). PRISMA and BioID disclose a motifs-based interactome of the intrinsically disordered transcription factor C/EBPalpha. iScience.

[13] Schmidt L., Heyes E., Grebien F. (2020). Gain-of-Function Effects of N-Terminal CEBPA Mutations in Acute Myeloid Leukemia. Bioessays.

[14] Dittmar G., Hernandez D.P., Kowenz-Leutz E., Kirchner M., Kahlert G., Wesolowski R., Baum K., Knoblich M., Hofstätter M., Muller A. (2019). PRISMA: Protein Interaction Screen on Peptide Matrix Reveals Interaction Footprints and Modifications- Dependent Interactome of Intrinsically Disordered C/EBPbeta. iScience.

[15] Leutz A., Pless O., Lappe M., Dittmar G., Kowenz-Leutz E. (2011). Crosstalk between phosphorylation and multi-site arginine/lysine methylation in C/EBPs. Transcription.

[16] Bararia D., Kwok H.S., Welner R.S., Numata A., Sárosi M.B., Yang H., Wee S., Tschuri S., Ray D., Weigert O. (2016). Acetylation of C/EBPalpha inhibits its granulopoietic function. Nat. Commun..

[17] Torcal Garcia G., Kowenz-Leutz E., Tian T.V., Klonizakis A., Lerner J., De Andres-Aguayo L., Sapozhnikova V., Berenguer C., Carmona M.P., Casadesus M.V. (2023). Carm1-arginine methylation of the transcription factor C/EBPalpha regulates transdifferentiation velocity. Elife.

[18] Cirovic B., Schönheit J., Kowenz-Leutz E., Ivanovska J., Klement C., Pronina N., Bégay V., Leutz A. (2017). C/EBP-Induced Transdifferentiation Reveals Granulocyte-Macrophage Precursor-like Plasticity of B Cells. Stem Cell Rep..

[19] Nguyen L.T., Zimmermann K., Kowenz-Leutz E., Lim R., Hofstätter M., Mildner A., Leutz A. (2024). C/EBPbeta-induced lymphoid-to-myeloid transdifferentiation emulates granulocyte-monocyte progenitor biology. Stem Cell Rep..

[20] Cheetham S.W., Faulkner G.J., Dinger M.E. (2020). Overcoming challenges and dogmas to understand the functions of pseudogenes. Nat. Rev. Genet..

[21] Ikuta K., Weissman I.L. (1992). Evidence that hematopoietic stem cells express mouse c-kit but do not depend on steel factor for their generation. Proc. Natl. Acad. Sci. USA.

[22] Li C.L., Johnson G.R. (1995). Murine hematopoietic stem and progenitor cells: I. Enrichment and biologic characterization. Blood.

[23] Eaves C.J. (2015). Hematopoietic stem cells: concepts, definitions, and the new reality. Blood.

[24] Rodrigues C.P., Shvedunova M., Akhtar A. (2021). Epigenetic Regulators as the Gatekeepers of Hematopoiesis. Trends Genet..

[25] Cenik B.K., Shilatifard A. (2021). COMPASS and SWI/SNF complexes in development and disease. Nat. Rev. Genet..

[26] Ohlsson E., Hasemann M.S., Willer A., Lauridsen F.K.B., Rapin N., Jendholm J., Porse B.T. (2014). Initiation of MLL-rearranged AML is dependent on C/EBPalpha. J. Exp. Med..

[27] Grebien F., Vedadi M., Getlik M., Giambruno R., Grover A., Avellino R., Skucha A., Vittori S., Kuznetsova E., Smil D. (2015). Pharmacological targeting of the Wdr5-MLL interaction in C/EBPalpha N-terminal leukemia. Nat. Chem. Biol..

[28] Pedersen T.Å., Kowenz-Leutz E., Leutz A., Nerlov C. (2001). Cooperation between C/EBPalpha TBP/TFIIB and SWI/SNF recruiting domains is required for adipocyte differentiation. Genes Dev..

[29] Muller C., Calkhoven C.F., Sha X., Leutz A. (2004). The CCAAT enhancer-binding protein alpha (C/EBPalpha) requires a SWI/SNF complex for proliferation arrest. J. Biol. Chem..

[30] Muller C., Alunni-Fabbroni M., Kowenz-Leutz E., Mo X., Tommasino M., Leutz A. (1999). Separation of C/EBPalpha-mediated proliferation arrest and differentiation pathways. Proc. Natl. Acad. Sci. USA.

[31] Wysocka J., Swigut T., Milne T.A., Dou Y., Zhang X., Burlingame A.L., Roeder R.G., Brivanlou A.H., Allis C.D. (2005). WDR5 associates with histone H3 methylated at K4 and is essential for H3 K4 methylation and vertebrate development. Cell.

[32] Schmidt L., Heyes E., Scheiblecker L., Eder T., Volpe G., Frampton J., Nerlov C., Valent P., Grembecka J., Grebien F. (2019). CEBPA-mutated leukemia is sensitive to genetic and pharmacological targeting of the MLL1 complex. Leukemia.

[33] Guarnaccia A., Tansey W. (2018). Moonlighting with WDR5: A Cellular Multitasker. J. Clin. Med..

[34] Song J.J., Kingston R.E. (2008). WDR5 interacts with mixed lineage leukemia (MLL) protein via the histone H3-binding pocket. J. Biol. Chem..

[35] Saber J., Rudnicki M.A. (2022). Carm1 and the Epigenetic Control of Stem Cell Function. Stem Cells Transl. Med..

[36] Torcal Garcia G., Graf T. (2021). The transcription factor code: a beacon for histone methyltransferase docking. Trends Cell Biol..

[37] Kowenz-Leutz E., Pless O., Dittmar G., Knoblich M., Leutz A. (2010). Crosstalk between C/EBPbeta phosphorylation, arginine methylation, and SWI/SNF/Mediator implies an indexing transcription factor code. EMBO J..

[38] Rosenbauer F., Koschmieder S., Steidl U., Tenen D.G. (2005). Effect of transcription-factor concentrations on leukemic stem cells. Blood.

[39] Dahl R., Walsh J.C., Lancki D., Laslo P., Iyer S.R., Singh H., Simon M.C. (2003). Regulation of macrophage and neutrophil cell fates by the PU.1:C/EBPalpha ratio and granulocyte colony-stimulating factor. Nat. Immunol..

[40] van der Lee R., Buljan M., Lang B., Weatheritt R.J., Daughdrill G.W., Dunker A.K., Fuxreiter M., Gough J., Gsponer J., Jones D.T. (2014). Classification of Intrinsically Disordered Regions and Proteins. Chem. Rev..

[41] Moses A.M., Hériché J.-K., Durbin R. (2007). Clustering of phosphorylation site recognition motifs can be exploited to predict the targets of cyclin-dependent kinase. Genome Biol..

[42] Holehouse A.S., Kragelund B.B. (2024). The molecular basis for cellular function of intrinsically disordered protein regions. Nat. Rev. Mol. Cell Biol..

[43] Christou-Kent M., Cuartero S., Garcia-Cabau C., Ruehle J., Naderi J., Erber J., Neguembor M.V., Plana-Carmona M., Alcoverro-Bertran M., De Andres-Aguayo L. (2023). CEBPA phase separation links transcriptional activity and 3D chromatin hubs. Cell Rep..

[44] Crowley P.B., Golovin A. (2005). Cation-pi interactions in protein-protein interfaces. Proteins.

[45] Fisher R.S., Elbaum-Garfinkle S. (2020). Tunable multiphase dynamics of arginine and lysine liquid condensates. Nat. Commun..

[46] Gallivan J.P., Dougherty D.A. (1999). Cation-interactions in structural biology. Chemistry.

[47] Wang Q., Li Z., Zhang S., Li Y., Wang Y., Fang Z., Ma Y., Liu Z., Zhang W., Li D. (2022). Global profiling of arginine dimethylation in regulating protein phase separation by a steric effect-based chemical-enrichment method. Proc. Natl. Acad. Sci. USA.

[48] Tilvawala R., Thompson P.R. (2019). Peptidyl arginine deiminases: detection and functional analysis of protein citrullination. Curr. Opin. Struct. Biol..

[49] Scheich C., Kummel D., Soumailakakis D., Heinemann U., Bussow K. (2007). Vectors for co-expression of an unrestricted number of proteins. Nucleic Acids Res..

